# Small molecule inhibitor regorafenib inhibits RET signaling in neuroblastoma cells and effectively suppresses tumor growth *in vivo*

**DOI:** 10.18632/oncotarget.22011

**Published:** 2017-10-24

**Authors:** Zhenghu Chen, Yanling Zhao, Yang Yu, Jonathan C. Pang, Sarah E. Woodfield, Ling Tao, Shan Guan, Huiyuan Zhang, Shayahati Bieerkehazhi, Yan Shi, Roma Patel, Sanjeev A. Vasudevan, Joanna S. Yi, Jodi A. Muscal, Guo-Tong Xu, Jianhua Yang

**Affiliations:** ^1^ Department of Ophthalmology, Shanghai Tenth People’s Hospital, Tongji University School of Medicine, Shanghai 200072, P. R. China; ^2^ Texas Children’s Cancer Center, Department of Pediatrics, Dan L. Duncan Cancer Center, Baylor College of Medicine, Houston, Texas 77030, USA; ^3^ Department of Biosciences, Weiss School of Natural Sciences, Rice University, Houston, Texas 77005, USA; ^4^ Division of Pediatric Surgery, Texas Children’s Hospital Department of Surgery, Michael E. DeBakey Department of Surgery, Dan L. Duncan Cancer Center, Baylor College of Medicine, Houston, Texas 77030, USA; ^5^ Department of Labour Hygiene and Sanitary Science, College of Public Health, Xinjiang Medical University, Urumqi, Xinjiang 830011, P.R. China; ^6^ Department of Pathology, University of Texas MD Anderson Cancer Center, Houston, Texas 77030, USA

**Keywords:** neuroblastoma, RET, regorafenib, chemotherapy, tyrosine kinase inhibitor

## Abstract

Neuroblastoma (NB), the most common extracranial pediatric solid tumor, continues to cause significant cancer-related morbidity and mortality in children. Dysregulation of oncogenic receptor tyrosine kinases (RTKs) has been shown to contribute to tumorigenesis in various human cancers and targeting these RTKs has had therapeutic benefit. RET is an RTK which is commonly expressed in NB, and high expression of RET correlates with poor outcomes in patients with NB. Herein we report that RET is required for NB cell proliferation and that the small molecule inhibitor regorafenib (BAY 73-4506) blocks glial cell derived neurotrophic factor (GDNF)-induced RET signaling in NB cells and inhibits NB growth both *in vitro* and *in vivo*. We found that regorafenib significantly inhibited cell proliferation and colony formation ability of NB cells. Moreover, regorafenib suppressed tumor growth in both an orthotopic xenograft NB mouse model and a *TH-MYCN* transgenic NB mouse model. Finally, regorafenib markedly improved the overall survival of *TH-MYCN* transgenic tumor-bearing mice. In summary, our study suggests that RET is a potential therapeutic target in NB, and that using a novel RET inhibitor, like regorafenib, should be investigated as a therapeutic treatment option for children with NB.

## INTRODUCTION

Derived from neural crest, neuroblastoma (NB) continues to be the most common extracranial solid tumor in children. Due to the heterogeneity of this disease, the clinical behaviors of NB spans from spontaneous regression or differentiation to an aggressive, therapy-resistant phenotype in patients [1]. Over the past decades, a combination criteria of various clinical and biologic prognostic markers leads to the classification of this pediatric cancer into low-, intermediate- and high-risk groups [2]. Currently, *MYCN* amplification, which correlates with poor prognosis and outcome, remains the best-characterized genetic marker of high-risk NB [3]. Despite the advances in understanding the genomic and biological characteristics of NB, new therapies are needed for children with high-risk NB, where the cure rates of those patients are still unsatisfyingly low [4].

Protein kinases are essential for cell function in almost every aspect. Of the 518 protein kinases identified, 385 members are categorized as protein-serine/threonine kinases, 90 as protein-tyrosine kinases, and 43 as tyrosine-kinase like proteins [5]. Among the 90 protein tyrosine kinases, 58 are further classified as receptor tyrosine kinases (RTKs) and the other 32 as non-receptor proteins. Tyrosine kinases, especially the RTKs, play a central role in mediating cell survival, proliferation, migration and differentiation [6]. Dysregulation of RTKs has been frequently associated with a variety of human cancers. A variety of US food and drug administration (FDA) approved drugs have inhibited these oncogenic RTKs and demonstrate significant anti-tumor effects [7, 8]. Notably, 244 of 518 protein kinase encoded genes map to disease loci or cancer amplicons as demonstrated by chromosomal mapping [5], indicating that those oncogenic protein kinases may be potential drug targets in cancer therapy. Therefore, it is essential to identify targetable oncogenic RTKs specific to NB and in the future, to treat NB patients by small molecules targeting those RTKs.

The *RET* proto-oncogene encodes a RTK that harbors three domains: an N-terminal extracellular domain with four cadherin-like repeats, a hydrophobic transmembrane domain with a cysteine-rich region, and a cytoplasmic tyrosine kinase domain [9]. RET is the tyrosine kinase receptor that interacts with the glial cell-derived neurotrophic factor (GDNF) family of ligands (GFLs) including: GDNF, neurturin (NRTN), persephin (PSPN) and artemin (ARTN) [10]. The GFL first binds to its specific co-receptor, the GDNF receptor-α family (GFRα1–4), to form a GFL–GFRα complex. The GFL and GFRα association leads to RET dimerization to form a GFL(2)-GFRα(2)-RET(2) heterohexamer complex that triggers the activation of multiple signaling pathways, including RAF/MEK/ERK and PI3K/AKT/mTOR signaling [11]. Activation of these signaling pathways results in cell survival, proliferation, migration, and invasion. Oncogenic gene fusions and activating mutations of RET have been identified and well documented as the driving force of tumorigenesis in several adult cancer types [12–14], however, no mutations of RET in NB have been found to date [15]. Evidence to suggest that RET could be a viable target in NB include the following: RET is involved in the development of the neural crest, as well as the ontogenesis of the enteric nervous system and kidney [11], RET is commonly expressed in NB tissues, and cell lines and RET-mediated signaling pathways are functional in NB [11, 16]. Yet, the role of RET in NB remains to be determined.

Regorafenib is an orally active multi-kinase inhibitor targeting RET, as well as other RTKs including VEGFR1/2/3, FGFR-1, KIT, PDGFR-β, TIE-2, and serine/threonine-specific protein kinases RAF-1 and B-RAF [17]. Regorafenib has shown efficacy in studies against several cancer types and is approved by FDA for the treatment of advanced gastrointestinal stromal tumors (GIST) and advanced metastatic colorectal cancer (mCRC) [18, 19]. In this paper, we explore targeting RET as a viable therapeutic strategy in NB. We first show that high expression of RET correlates with poor outcome in NB patients in the SEQC-498-RPM data set. In addition, regorafenib suppresses NB growth both *in vitro* and *in vivo*. Regorafenib shows cytotoxic effects on NB cells and blocks GDNF-induced PI3K/AKT/mTOR signaling *in vitro*. Importantly, regorafenib potently inhibits tumor growth in both an orthotopic xenograft NB mouse model and a transgenic NB mouse model. Moreover, regorafenib treatment prolongs the lifespan of *TH-MYCN* transgenic tumor-bearing mice dramatically. Overall, our study suggests that RET is a therapeutic target in NB and use of RET inhibitor regorafenib may be a novel, effective treatment strategy for NB patients.

## RESULTS

### High expression of RET is associated with poor outcome in NB patients and RET is required for NB cell proliferation

**Figure 1:**
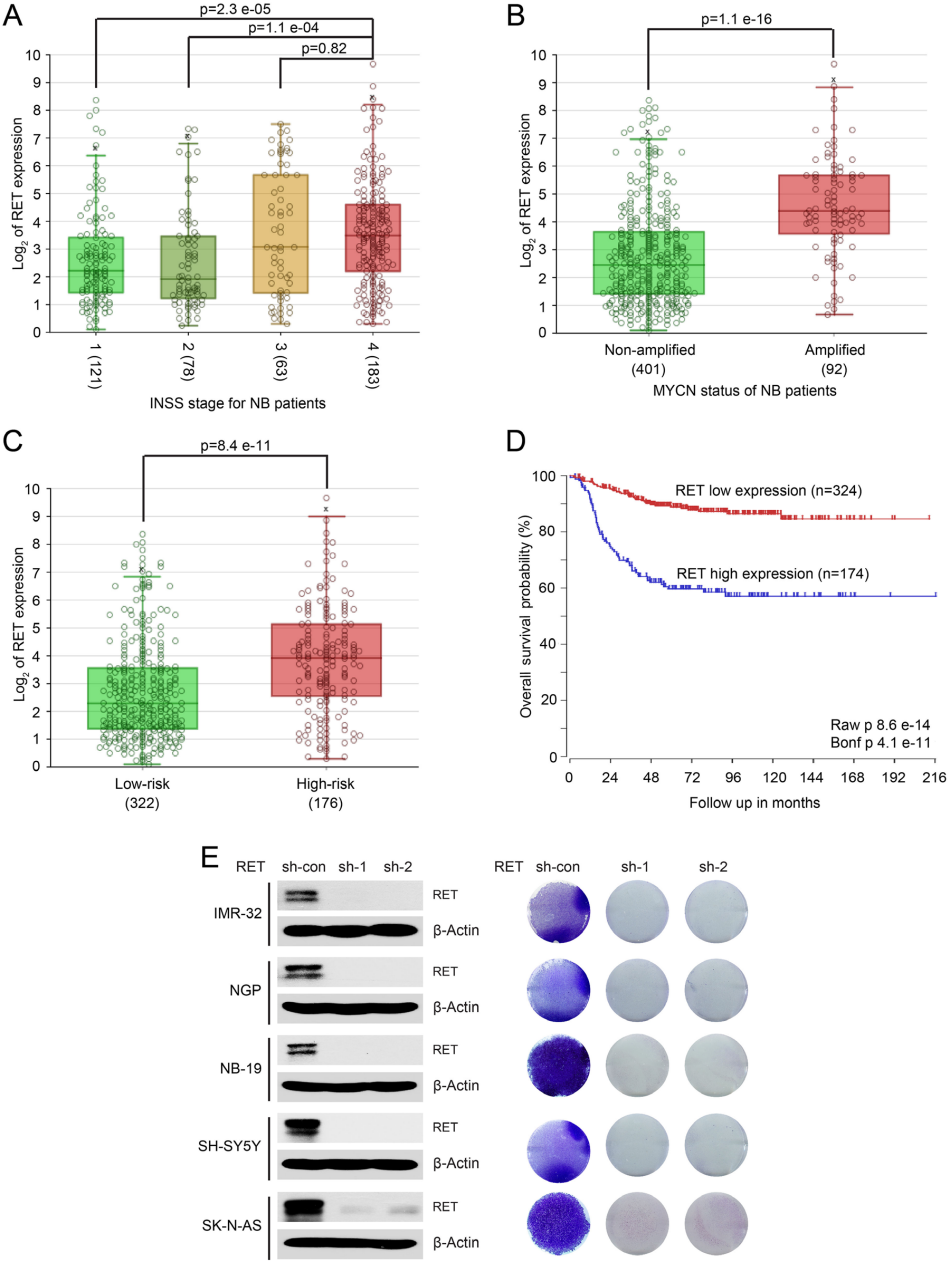
High expression of RET predicts poor outcome in patients with NB A large patient cohort (498 patients, SEQC-498-RPM data set) with annotated clinical and long term follow up data was used to analyze the outcomes in patients with NB. **(A)** Analysis of RET expression in NB patients from stage 1 to metastatic stage 4 demonstrates higher levels of *RET* in stage 4 metastatic subgroup compared with other groups (*P* values were calculated by Student T-test). High expression of RET correlates with *MYCN* amplification status **(B)**, high-risk disease **(C)**, and lower long-term survival probability **(D)** in patients with NB. This Kaplan–Meier survival curve was generated (*P* <0.001) comparing tumors with high (n = 174) versus low (n = 324) expression of RET. **(E)** RET knockdown decreased cell proliferation in a panel of NB cells. Protein immunoblotting analysis demonstrated RET knockdown in IMR-32, NGP, NB-19, SH-SY5Y and SK-N-AS cells by lenti-virus-mediated expression of shRNAs and anchorage-dependent cell proliferation images of those NB cells were shown.

To determine whether RET has prognostic value in NB, we evaluated the clinical significance of RET expression in patients with NB. RNA samples from a cohort of 498 NB patients were obtained and the same set of samples were profiled with RNA-Seq platform (R2: Genomics Analysis and Visualization Platform (http://r2.amc.nl). Based on that RNA-Seq results, data analysis with annotated clinical data of the SEQC-498-RPM data set demonstrates that RET is expressed in NB patients of all four International Neuroblastoma Staging System Committee (INSS) stages, and RET has higher expression levels in stage 4 metastatic subgroup compared with other groups (Figure 1A). Moreover, high expression of RET is associated with *MYCN* amplification (92/498 patients were amplified), high risk disease (176/498 patients were identified as “high-risk”) and worse long-term overall survival rates (57% survival in patients whose tumors had high RET expression versus 85% survival in patients whose tumors had low RET expression, *P* < 0.001) (Figure 1B-1D). These data suggest that RET may play a critical role in NB tumorigenesis and that overexpression of RET may contribute to NB tumor progression.

RET was expressed in a subset of NB cell lines, and knocking down RET by short hairpin RNAs (shRNAs) significantly inhibited cell proliferation in all of the NB cell lines tested (Figure 1E). These findings indicate that RET is important for NB cell proliferation and that targeting RET may achieve better outcomes for NB patients.

### Small molecule inhibitor regorafenib abrogates RET-mediated PI3K/AKT/mTOR signaling in NB cells

The PI3K/AKT/mTOR signaling pathway is a critical effector for cell proliferation and tumor progression [20–22]. Inhibition of this pathway by small molecule inhibitors showed significant anti-tumor effect on NB [23, 24]. The phosphorylation of RET at Y1062 is responsible for the activation of downstream PI3K/AKT/mTOR signaling that is important for NB progression [25, 26]. Small molecule inhibitor regorafenib has been suggested to be a novel RET inhibitor *in vitro* [17], and it exhibits anti-tumor effects in various cancer types [27, 28]. Thus, we hypothesized that regorafenib might be able to block RET-mediated PI3K/AKT/mTOR signaling pathway in NB.

**Figure 2:**
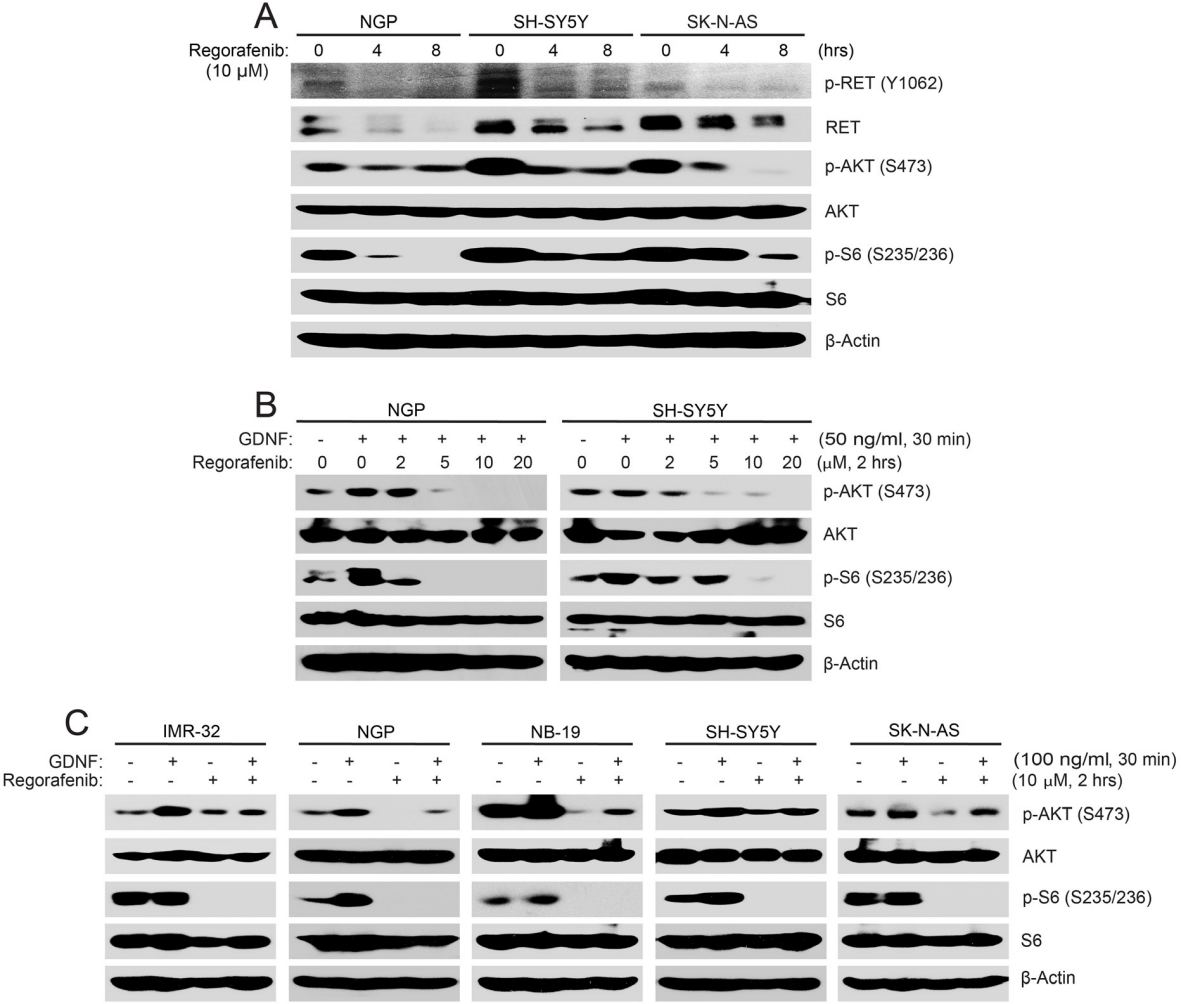
Regorafenib abrogates RET-mediated PI3K/AKT/mTOR signaling in NB cells **(A)** NGP, SH-SY5Y, and SK-N-AS cells were treated with 10 μM of regorafenib for 0–8 hrs, and treated cells were harvested at the end of treatment, subjected to SDS-PAGE, and immunoblotted with the indicated antibodies. **(B, C)** NGP and SH-SY5Y cells (B) or IMR-32, NGP, NB-19, SH-SY5Y and SK-N-AS cells (C) were starved with serum-free medium and then treated with increasing doses of regorafenib for 2 hrs prior to GDNF stimulation. Cell pellets were collected and analyzed by immunoblotting. β-Actin was used as a loading control for whole cell extracts in all samples.

To test this hypothesis, three NB cell lines (NGP, SH-SY5Y and SK-N-AS) were treated with regorafenib and phosphorylation of RET and activity of downstream signaling were examined. As shown in Figure 2A, we found that regorafenib diminished the phosphorylation of p-RET (Y1062) and inhibited the downstream phosphorylation of p-AKT (S473) and p-S6 (S235/236) in all of the three NB cell lines tested (Figure 2A).

GDNF and its co-receptor GFRα1 can form a complex with RET and that leads to the activation of RET and its downstream signaling pathways [25]. To test if the effects of regorafenib on NB cells results from inhibition of the RET-mediated signaling pathways, we performed a GDNF stimulation assay. Cells were starved in serum-free medium for 16 hrs before being exposed to various doses of regorafenib, followed by GDNF ligand stimulation. Regorafenib blocked GDNF-induced phosphorylation of p-AKT (S473) and p-S6 (S235/236) in all five NB cell lines tested (Figure 2B, 2C). Taken together, these data illustrate that regorafenib significantly inhibited RET-mediated PI3K/AKT/mTOR signaling pathway in NB cells.

### Regorafenib suppresses cell viability of NB *in vitro*

**Figure 3:**
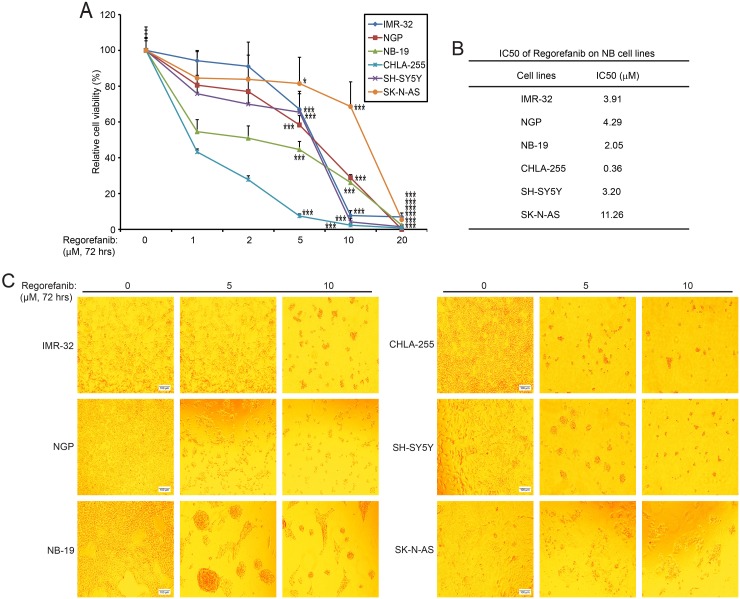
RET inhibitor regorafenib shows cytotoxic effects on NB cell lines **(A)** Six NB cell lines (IMR-32, NGP, NB-19, CHLA-255, SH-SY5Y, and SK-N-AS) were treated with the indicated concentrations of regorafenib for 72 hrs. Cell viability was then measured by CCK-8 assays and data were represented as % vehicle ± S.D. *P* <0.05 (^*^), or *P* <0.001 (^***^) (Student’s t-test, two-tailed). **(B)** IC50s of regorafenib on each NB cell line was calculated by using Prism 5.0, based on the data collected in the cell viability assays. **(C)** Morphologic changes of the six NB cell lines treated with increasing concentrations of regorafenib for 72 hrs.

Since RET is essential for NB cell proliferation, we then hypothesized that its small molecule inhibitor regorafenib would have an anti-tumor effect on NB. We assessed the cytotoxic effect of regorafenib on six NB cells including IMR-32, NGP, NB-19, CHLA-255, SH-SY5Y, and SK-N-AS. In a dose-dependent manner, regorafenib significantly suppressed cell viability in all of the NB cell lines tested (Figure 3A). IC50 values of regorafenib on the six NB cell lines were estimated and were all lower than 12 μM (Figure 3B). The median IC50 value for the six cell lines was 3.56 μM, with a range from 0.36 μM (CHLA-255) to 11.26 μM (SK-N-AS). The cytotoxic effect of regorafenib on these cells was confirmed by examining the cell morphological changes seen with treated cells (Figure 3C). These data show that small molecule inhibitor regorafenib is cytotoxic to NB cells in a dose dependent manner.

### Regorafenib attenuates the colony formation ability of NB cells

**Figure 4:**
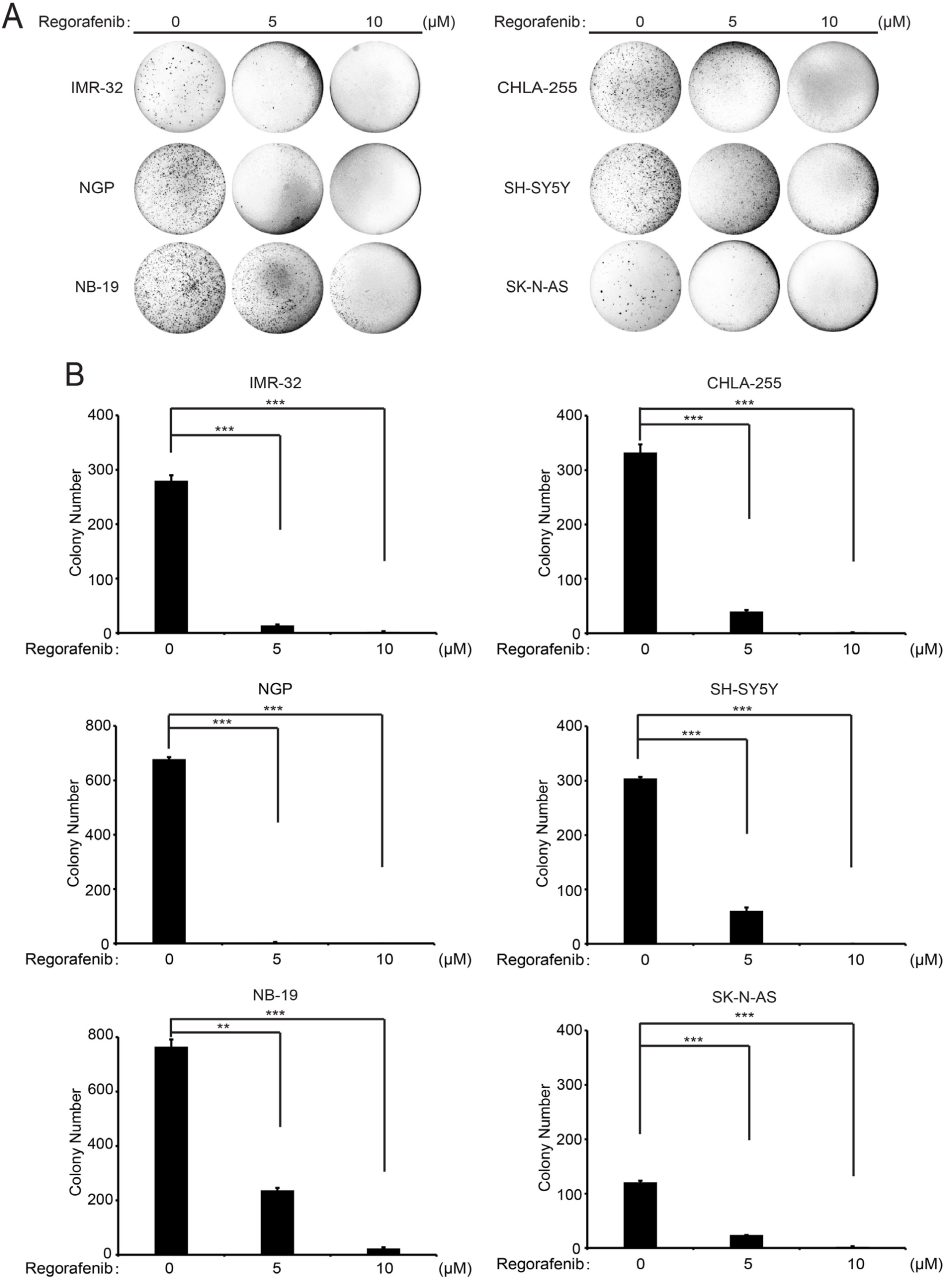
Regorafenib suppresses anchorage-independent growth of NB cell lines **(A)** A panel of six NB cell lines were seeded in six-well plates with soft agar. The cells were then treated with indicated concentrations of regorafenib and grown for two to three weeks. Cells were stained with crystal violet and photographed. **(B)** Cell colonies from (A) were counted and colony numbers were represented as % vehicle ± S.D. *P* <0.01 (^**^) or *P* <0.001 (^***^) (Student’s t-test, two-tailed).

The ability to form colonies in soft agar cultures in an anchorage-independent manner is one of the most distinctive properties of cancer cells. To evaluate whether regorafenib impacted the colony formation ability of NB cells, such soft agar assays were performed with NB cells treated with regorafenib. Regorafenib treatment led to dramatically decreased colony numbers of the six treated NB cell lines compared with the control groups, indicating that regorafenib suppresses the ability to form colonies of NB cells (Figure 4A, 4B). These results demonstrate that regorafenib significantly inhibits anchorage-independent growth of NB cell lines.

### Regorafenib inhibits tumor growth in an orthotopic xenograft NB mouse model by blocking the PI3K/AKT/mTOR signaling

**Figure 5:**
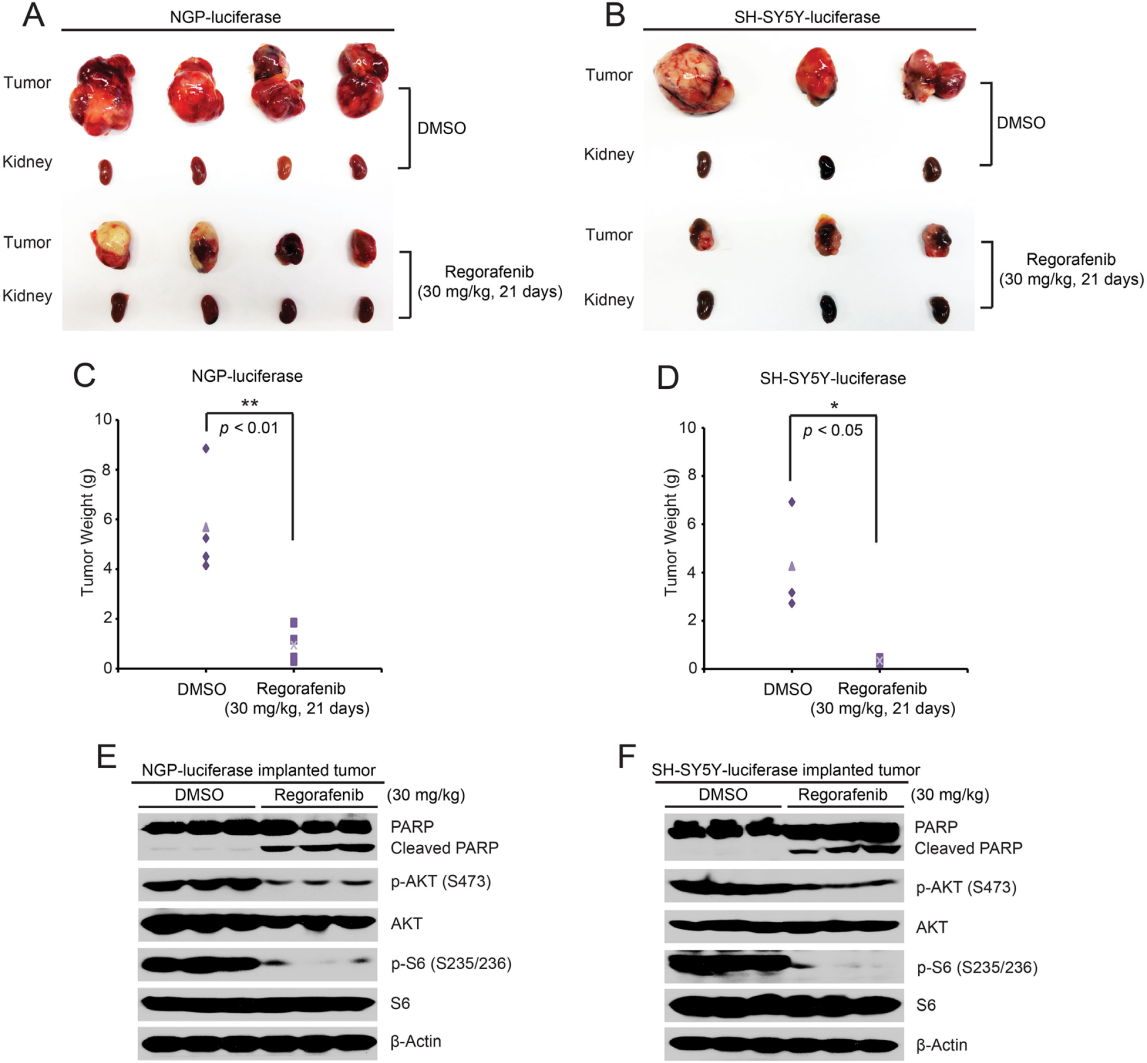
Regorafenib inhibits tumor growth in an orthotopic xenograft NB mouse model **(A, B)** Photos of NGP (A) and SH-SY5Y (B) xenografted tumors and corresponding kidney controls from the DMSO control group and the regorafenib treated group (30 mg/kg) were taken at the end of treatment (21 days). **(C, D)** NGP-derived tumor weights (C) from control (N=4) and treatment (N=4) groups and SH-SY5Y-derived tumor weights (D) from control (N=3) and treatment (N=3) groups, were presented. *P* <0.05 (^*^) and *P* <0.01 (^**^) (Student’s t-test, two-tailed). **(E, F)** Mice bearing NGP and SH-SY5Y xenografted tumors were treated with either regorafenib (30 mg/kg) or an equal volume of DMSO by i.p. injection daily for two days and the mice were sacrificed and the tumors were harvested and lysed for immunoblotting with the indicated antibodies. β-Actin was measured as a loading control.

To test the *in vivo* efficacy of regorafenib against NB, an orthotopic xenograft NB mouse model was established by using NGP and SH-SY5Y cell lines. The grouped mice were treated with either regorafenib (30 mg/kg) or an equal volume of dimethylsulfoxide (DMSO) by intraperitoneal (i.p.) injection daily for 21 days. At the end of treatment, tumor-bearing mice in each group were sacrificed and tumors were photographed and weighed. Compared with the control group, the regorafenib treated group displayed much smaller tumor in both the NGP and SH-SY5Y xenografted NB mouse models (Figure 5A-5D).

To further explore the molecular mechanism of the inhibitory effects of regorafenib on the orthotopic xenograft NB mouse model, another set of NGP-luciferase or SH-SY5Y-luciferase xenografted mice grouped by bioluminescent imaging values were treated with either regorafenib (30 mg/kg) or an equal volume of DMSO by i.p. injection daily for two days. Consistent with the *in vitro* data, regorafenib dramatically eliminated the phosphorylation of p-AKT (S473) and p-S6 (S235/236) in the tumor cells from the treatment group (Figure 5E, 5F). In addition, regorafenib significantly induced cell death in the tumor cells of both NGP and SH-SY5Y xenografted tumors as shown by induction of PARP cleavage (Figure 5E, 5F). Together, these results demonstrate that regorafenib alone significantly inhibited tumor growth and induced tumor cell death in an orthotopic xenograft NB mouse model.

### Regorafenib inhibits tumor development and prolongs survival in a *TH-MYCN* transgenic NB mouse model

**Figure 6:**
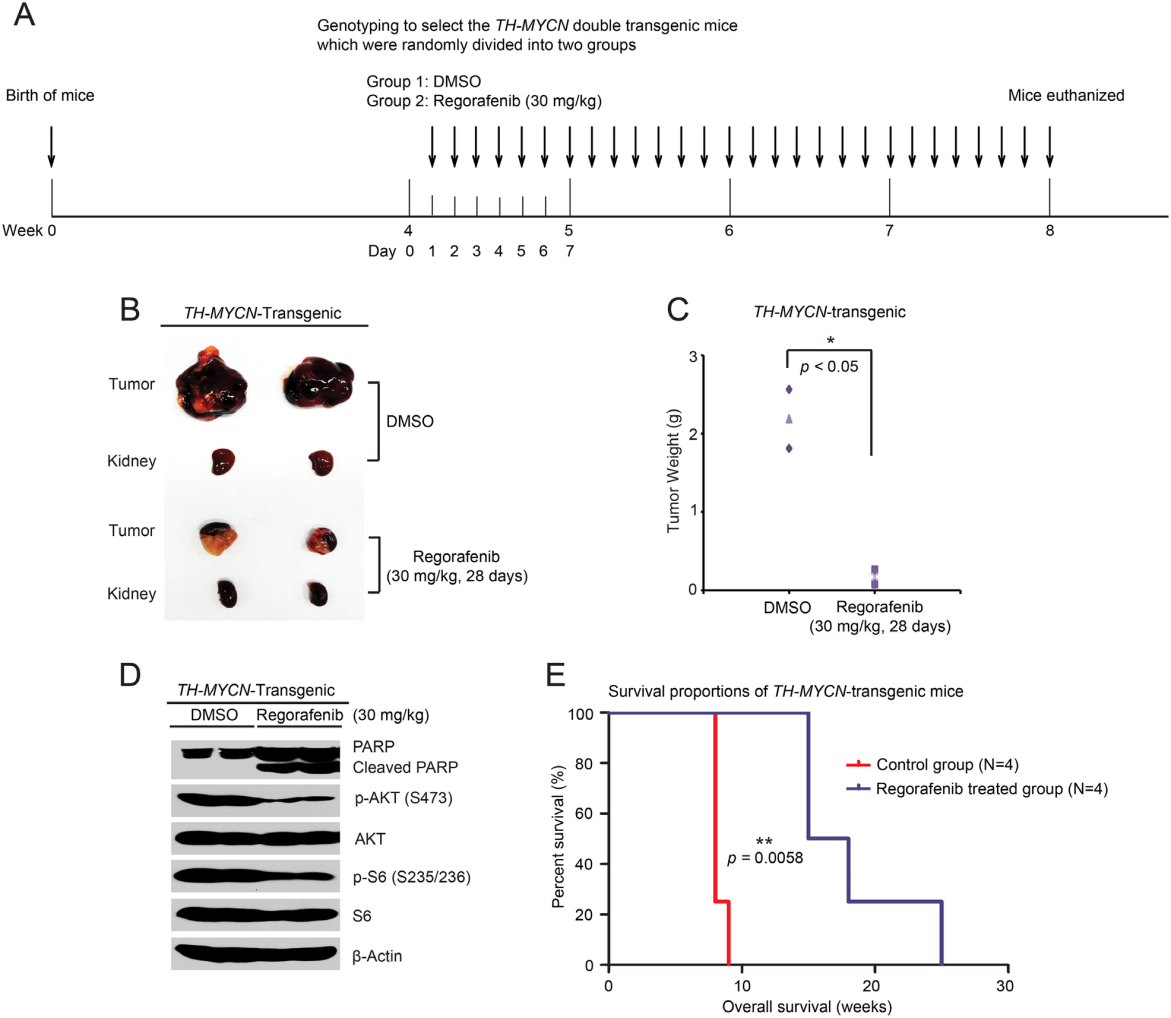
Regorafenib prevents tumor development and improves overall survival rate in the *TH-MYCN* transgenic NB mouse model **(A)** Treatment strategy schema for this study. **(B, C)** Pictures of tumors and corresponding kidneys at the end of treatment (B) and tumor weights (C) shown in the control (N=2) and treated (N=2) groups. Experiments were performed with two mice in each group. *P* <0.05 (^*^) (Student’s t-test, two-tailed). **(D)** Six-week old homozygous *TH-MYCN* transgenic mice were treated with either 30 mg/kg of regorafenib or an equal volume of DMSO by i.p. injection daily for two days. Then tumors were harvested and lysed for immunoblotting with the indicated antibodies. β-Actin was detected as a loading control. **(E)** Homozygous *TH-MYCN* transgenic mice from control (N=4) and treatment (N=4) groups were treated as in (A), and then tumors were removed after four weeks of treatment. Death dates of the tumor bearing mice were collected and the Kaplan-Meier survival curves were generated by using Prism 5.0 software. *P* <0.01 (^**^) (MCLR test).

To better simulate human NB, the *TH-MYCN* genetically-engineered murine NB model was established. Homozygous *TH-MYCN* transgenic mice have a normal immune system and develop tumors that mimic the development of human NB, and this mouse model is widely used in NB research [29, 30]. To assess the anti-tumor efficacy of regorafenib in a *TH-MYCN* transgenic NB mice, 4-week-old homozygous *TH-MYCN* transgenic mice were treated daily for 28 days (Figure 6A). Consistent with the data obtained from the orthotopic NB mouse model, regorafenib treatment resulted in markedly decreased tumor development in the *TH-MYCN* transgenic NB mouse model (Figure 6B, 6C). To probe the mechanism of this anti-tumor effect, another set of the *TH-MYCN* transgenic mice were treated daily for two days with 30mg/kg regorafenib or an equal volume of DMSO. Phosphorylation of p-AKT (S473) and p-S6 (S235/236) in regorafenib-treated tumor sections were markedly decreased compared with the control group (Figure 6D). Similar to the results from the orthotopic NB mouse model, regorafenib treatment induced PARP cleavage in the treated *TH-MYCN* transgenic NB mouse model, suggesting a mechanism of tumor cell death (Figure 6D).

To evaluate the effect of regorafenib on overall survival *in vivo*, a total of eight homozygous *TH-MYCN* transgenic mice were randomly divided into two groups. Regorafenib was administered as shown in Figure 6A, but without sacrificing after the four-week treatment. The survival data was collected and analyzed. Survival was significantly prolonged in the regorafenib-treated group compared with the control group (Figure 6E). These data show that regorafenib inhibited NB tumor growth by blocking the PI3K/AKT/mTOR signaling *in vivo*.

## DISCUSSION

Global activation of the RET signaling pathway has been implicated in NB and RET signaling is indispensable in GDNF-induced enhancement of non-adherent proliferation of NB-39-nu cells, which showed high expression and elevated phosphorylation of RET, suggesting that the RET signaling pathway may contribute to the metastasis of NB [31]. As NB is derived from the neural crest lineage, high expression of RET in neural crest-derived cells suggests that RET may play a role in the tumorigenesis of NB [11]. Previous studies have shown that RET is activated in retinoic acid (RA)-induced differentiation of NB cells and is a key player in this process of differentiation [32–35]. However, here in our study, we found that knocking down RET strongly suppressed the cell proliferation in NB cells, suggesting that RET is essential for NB cell growth (Figure 1E). Moreover, RET was widely expressed in NB patients with tumors of different stages (Figure 1A) and high expression of the RET tyrosine kinase was associated with poor outcome of these patients (Figure 1D). These data suggest that RET likely plays an oncogenic role in NB development.

As a multi-kinase inhibitor, regorafenib targets a variety of RTKs including RET, VEGFRs, FGFR-1 and PDGFR-β, and others, in which regorafenib is most potent against RET [17]. In contrast to the previously discovered small molecule inhibitors, regorafenib has a broad spectrum and relatively low IC50s for many of the RTKs. Regorafenib has demonstrated toxic effects on a variety of pediatric solid tumor cell lines including NB cells [36]. Regorafenib also has been reported to suppress the angiogenesis and induce apoptosis in an orthotopic adrenal NB mouse model by inhibiting PDGFR-β signaling [36]. Here in this study we report a novel working mechanism of regorafenib on NB. We found that regorafenib blocked GDNF-induced RET signaling in NB cells. Furthermore, regorafenib exerts potent anti-tumor effects in an orthotopic xenograft NB mouse model. Regorafenib also inhibited tumor growth in a *TH-MYCN* transgenic NB mouse model, and regorafenib treatment resulted in dramatically improved survival of the *TH-MYCN* transgenic tumor-bearing mice. Overall, our study reveals that regorafenib is a potent RET inhibitor in NB and that RET is a potential therapeutic target in this devastating pediatric malignancy.

Multiple RET inhibitors have been studied in the laboratory and in the clinic. Vandetanib, the first generation RET inhibitor, has shown activity in patients with advanced non-small cell lung cancer harboring RET rearrangements [37]. However, there is a low response rate of vandetanib alone in lung cancer [38] and vandetanib failed to show a significant advantage to patients in a large retrospective study [39]. The multi-targeted kinase inhibitor ponatinib showed promising preclinical activity in thyroid carcinoma cells and in a RET-driven medullary thyroid carcinoma mouse model [40]. However, side effects were observed when ponatinib was used to treat patients with chronic myeloid leukemia (CML) [41]. Another RET inhibitor cabozantinib is effective against a subset of NB cells *in vitro* by decreasing RET and ERK phosphorylation and cabozantinib inhibits tumor growth *in vivo* [42], suggesting that targeting RET is a promising treatment strategy in NB. Other RET inhibitors, sorafenib and sunitinib demonstrated anti-tumor effects on KIF5B-RET transformed cells [43, 44]. Still, the safety and clinic efficacy of those inhibitors needs to be further determined.

The side effects of regorafenib in patients with mCRC and GIST include hand-foot skin reactions (HFSR), diarrhea, hypertension and fatigue [45]. However, in our study, no obvious side effects were observed when the mice were treated with regorafenib. Other targeted RTKs of regorafenib, including VEGFRs, FGFRs, PDGFRs, and TIE-2, control the maturation of immature vessels in tumors and promotes tumor angiogenesis [46, 47]. The activation of those RTK-mediated signaling pathways in the tumor microenvironment play a critical role in driving tumorigenesis [48, 49]. Therefore, the anti-tumor effects of regorafenib on NB may due to its combinational inhibition of RET and PDGFR-β signaling in NB cells, and the inhibition of tumor angiogenesis in the tumor microenvironment in tumor associate cells. Despite the many advantages of regorafenib in cancer therapy and its shown efficacies, the exact mechanism underlying the anti-tumor efficacy of regorafenib needs to be further elucidated. In addition, because there is no RET specific inhibitor available to date, we are unable to test the effects of RET single agent on NB. Therefore, the development of novel RET inhibitors could assist in investigating the precise role of RET in NB.

In summary, our study reveals the oncogenic role of RET and the *in vitro* and *in vivo* efficacy of RET inhibitor regorafenib in NB, especially in the *TH-MYCN* transgenic mice. Regorafenib inhibits NB growth both *in vitro* and *in vivo* by blocking RET-mediated PI3K/AKT/mTOR signaling. Therefore, clinical trials of regorafenib should be pursued in children with NB to further explore the potential benefit of this drug.

## MATERIALS AND METHODS

### *RET* expression analysis in NB cohorts

Analysis of *RET* expression was performed by using the R2: microarray analysis and visualization platform (http://r2.amc.nl). The Neuroblastoma SEQC-498-RPM patient cohort [50] consists of 498 patients with *RET* gene expression data, tumor stages, patient risk information, and annotated overall survival. The long-term survival curve among high and low expression of*RET* was created by Kaplan–Meier analysis in R2 database.

### Antibodies and reagents

Multi-kinase inhibitor regorafenib was purchased from Selleckchem (S1178) (Selleckchem, Houston, TX, USA). Anti-β-Actin (A2228) antibody was purchased from Sigma (Sigma-Aldrich Corp, St. Louis, MO, USA). Anti-p-RET (Y1062) (SC-20252) antibody was from Santa Cruz Biotechnology (Santa Cruz Biotechnology, Dallas, TX, USA). Anti-RET (14556S), anti-PARP (9532S), anti-p-AKT (Ser473) (4060S), anti-AKT (9272S), anti-p-S6 (S235/236) (4858S) and anti-S6 (2217S) primary antibodies, as well as anti-Rabbit (7074S) and anti-mouse (7076S) secondary antibodies were purchased from Cell Signaling Technology (Cell Signaling Technology, Danvers, MA, USA).

### Cell lines and cell culture

The three *MYCN*-amplified (IMR-32, NGP and NB-19) and three MYCN-non-amplified (CHLA-255, SH-SY5Y and SK-N-AS) human NB cell lines were cultured in RPMI Medium 1640 (RPMI) (Lonza, Walkersville, MD, USA), which was complemented with 10% (v/v) heat-inactivated Fetal Bovine Serum (FBS) (SAFC Biosciences, Lenexa, KS, USA), 100 μg/mL streptomycin, and 100 units/mL penicillin. All cells were kept in a humidified incubator at 37°C and with 5% CO_2_. Cells used to perform the experiments were under exponential growth conditions. pcDNA3 luciferase expression plasmids were transfected into NGP and SH-SY5Y cells to generate the NGP-luciferase and SH-SY5Y-luciferase cells with stable luciferase expression. The typical stable cell lines were established after 10 days of 800 μg/ml or 400 μg/ml G418 (Enzo Life Sciences, Farmingdale, NY, USA) selection.

### Cell viability assay

Cell Counting KIT-8 assay (CCK-8, WST-8[2-(2-methoxy-4-nitrophenyl)-3-(4-nitrophenyl)-5-(2,4-disulfophenyl)-2 H-tetrazolium, monosodium salt]) (Dojindo Laboratories, Rockville, MA, USA) was performed as previously described [51, 52]. Briefly, cells were seeded and grown in 96-well plates from 1 × 10^4^ cells/well. After 24 hours (hrs) of incubation, the media were changed and increasing doses of regorafenib were added to the wells. The cells were then incubated at 37°C for 72 hrs. Then a mixture of 10 μL of CCK-8 and 190 μL of RPMI with 10% FBS was added into each well. The absorbance of each well was detected by using a microplate reader at 450 nm after one hour. Each experiment was performed in six replicates and the background reading of the media was subtracted from each well to standardize the reading results.

### Cell imaging

A total of six NB cell lines were seeded in 96-well plates from 1 × 10^4^ cells/well. After 72 hrs of treatment with the indicated concentrations of regorafenib or equal volumes of DMSO, cell morphologies were captured using an optical microscope. Each result was performed in triplicate.

### Generation of gene knockdown NB cell lines

The TRC2 lenti-viral vector was used to generate shRNA plasmids for human *RET* gene. The following target sequences were selected:

RET-sh-control: 5'- CGTCTTTTCGGACTTAGAGAG -3'

RET-sh-1: 5'-AATTTCCCATGCATTTACTAG-3'

RET-sh-2: 5'-AAGAGGAGAGACTACTTGGAC-3'.

The sequences of these plasmids were further confirmed by DNA sequencing. The TRC2 vector bearing the above sequences and four packaging vectors (Hgpm, Tat-1b, Rev-1b, and VSVG) were then transfected into the packaging cell line HEK293T (2.5 × 10^6^ cells/dish). The viral supernatants were collected 48 hrs later. Five NB cell lines (IMR-32, NGP, NB-19, SH-SY5Y and SK-N-AS) were seeded in six-well plates (2∼5 × 10^5^ cells/well) and incubated with the viral supernatants and 4 μg/ml polybrene. The viral transduced NB cells selected by 0.5 μg/ml puromycin and the stable cell lines were established after the selection. The selected cells were then used to perform the following experiments.

### Anchorage-dependent cell proliferation assay

Selected NB cells were seeded in six-well plates (1 × 10^5^ cells/well) and grown for one to two weeks. Then cells were washed with cold PBS three times and fixed in cold methanol for 15 minutes (min) at room temperature (RT, 25°C). The fixed cells were stained with 0.05% crystal violet for 10 min at RT, washed with tap water twice, and air dried at RT. Next, images were obtained of stained cells and scanned. Each assay was performed in triplicate.

### Colony formation assay

The colony formation assay was performed as previously described [53, 54]. Briefly, a 5% (w/v) base agar (214220, Difco Laboratories, Detroit, MI, USA) was added into distilled water and was then autoclaved for 50 min. Next, the mixture was cooled down in a 56°C water bath. For the bottom agar layer, 2 mL of the 0.5% agar/RPMI solution was added to each well of the six-well plates and the solution was cooled to semi-solid status. The top agar layer consisted of 1.5 ml 0.3% agar and the prepared NB cell lines were counted and added to the mixture at 1 × 10^4^ cells/well. The indicated concentrations of regorafenib was added to the wells 24 hrs later. Cells were maintained at 37°C for two to three weeks, then stained with 500 μL of 0.005% crystal violet (C3886, Sigma). Four hours later, the plates were photographed by the VersaDoc Imaging System (Bio-Rad Laboratories, Hercules, CA, USA) and colonies were counted by using Quantity One software (Bio-Rad Laboratories, Hercules, CA, USA). All the assays were performed in triplicate.

### Protein immunoblotting

The experiments were performed as described previously [55–57]. Briefly, at the end of the treatment, cells were washed with ice cold PBS twice and lysed for 30 min at 4°C in cooled RIPA buffer (50 mM Tris-HCl at pH 7.4, 150 mM NaCl, 1 mM EDTA, 1% NP-40, 0.25% sodium deoxycholate, 1 mM phenylmethylsulfonyl fluoride, 1 mM benzamidine, 10 μg/mL leupeptin, 1 mM dithiothreitol, 50 mM sodium fluoride, 0.1 mM sodium orthovanadate, and phosphatase inhibitor cocktail 2 and 3 (p5726 and p0044, Sigma)). After centrifuging at 13,000 rpm for 15 min, the supernatants were used as loading samples. Bradford reagent (Bio-Rad Laboratories, Hercules, CA, USA) was used to measure protein concentrations and each sample was mixed in a 3:1 ratio (v/v) with 4× loading buffer. The mixture was then heated at 100°C for 7 min. Loading samples were then separated by SDS-PAGE, transferred to polyvinylidence fluoride (PVDF) membranes (BioRad), blocked with 5% milk or BSA for one hour at RT, and incubated with the recommended dilutions of indicated primary antibodies overnight at 4°C. Anti-mouse or rabbit secondary antibodies conjugated with horseradish peroxidase were incubated with the membranes at RT for one hour. Chemiluminescent visualization was detected by using the ECL-Plus Western detection system (GE Health Care, Buckinghamshire, UK). β-Actin was used as a loading control for whole cell extracts in all groups.

### Anti-tumor efficacy in an orthotopic xenograft NB mouse model

The orthotopic mouse model of NB was established by orthotopically implanting NB cells as described previously [58–60]. Five to six-week-old female athymic NCR nude mice were purchased from Taconic (Taconic, Hudson, NY, USA) and maintained under barrier conditions. Briefly, a transverse incision was created in the left flank of the nude mice and 1.5 × 10^6^ human luciferase-transduced NGP or SH-SY5Y cells were then injected into the left renal capsule of the nude mice surgically. The tumor cells were then allowed to grow for two weeks and bioluminescent images of the tumors in the injected mice were captured and analyzed.

Tumor bearing mice of the two injected cell lines were standardized by a threshold of 1 × 10^6^ total flux (p/s) and the mice of similar tumor size were randomly and divided into two groups: a DMSO control group and a regorafenib treatment group. Each group contained three (SH-SY5Y-luciferase cells injected) or four (NGP-luciferase cells injected) mice, respectively. After treatment with regorafenib at 30 mg/kg or an equal volume of DMSO by intraperitoneal (i.p.) injection once daily for 21 days, all mice were sacrificed and the tumors, together with the right kidneys were harvested, weighed and photographed.

For protein immunoblotting, the NGP-luciferase or SH-SY5Y-luciferase implanted mice with similar tumor sizes were randomly divided into two groups and treated with either DMSO or regorafenib (30 mg/kg by i.p. injection) daily for two days. Two days later, the mice were sacrificed and the tumors were harvested and lysed for immunoblotting. All mice were handled according to protocols approved by the Institutional Animal Care and Use Committee of the Baylor College of Medicine.

### Anti-tumor efficacy in the *TH-MYCN* transgenic NB mouse model

To evaluate treatment efficacy, homozygous *TH-MYCN* transgenic mice were identified by genotyping PCR as previously described [61]. Four-week-old homozygous *TH-MYCN* transgenic mice were divided randomly into two groups. 30 mg/kg regorafenib or an equal volume of DMSO was administered daily by i.p. injection to the grouped mice for 28 days. At the end of the treatment the mice were sacrificed and the tumors of each group were photographed and weighed.

To analyze protein expression by immunoblotting assay, homozygous *TH-MYCN* transgenic mice of the same age were randomly divided into two groups (N=2 for each group) and treated with either regorafenib (30 mg/kg by i.p. injection) or an equal volume of DMSO daily for two days. Then the mice were sacrificed and the tumors were harvested and lysed for immunoblotting.

To evaluate survival, the identified homozygous *TH-MYCN* transgenic mice with the same age were divided into control and treatment groups (N=4 for each group) and 30 mg/kg regorafenib or an equal volume of DMSO was administered daily by i.p. injection to the mice for 28 days. After that, we stopped drug treatment and let the mice grow naturally. Death dates of the grouped mice were collected and survival curves were determined using the Kaplan-Meier method. All mice were handled according to protocols approved by the Institutional Animal Care and Use Committee of the Baylor College of Medicine.

### Statistical analysis

The statistical significance was determined by the two-tailed Student’s t-test for each *in vitro* and *in vivo* orthotopic xenograft NB mouse model assay, comparing the control and drug treated groups. Student’s t-test was also used to determine the statistical significance of the data from NB patient among different groups. To compare the survival between the groups test of *TH-MYCN* transgenic mice, the statistical significance was determined by MCLR (Mantel-Cox, log-rank) test using Prism 5.0 software. Each assay was performed at least twice and the representative results were presented in the figures below. *P* <0.05 was considered to be statistically significant in all assays.
